# *Notes from the Field*: Identification of Tourists from Switzerland Exposed to Rabies Virus While Visiting the United States — January 2018

**DOI:** 10.15585/mmwr.mm6716a5

**Published:** 2018-04-27

**Authors:** Emily G. Pieracci, Danielle Stanek, Daniel Koch, Katrin S. Kohl, Jesse D. Blanton, Terri Harder, Marian O’Brien, Hugo Leon, Pam Colarusso, Brittany Baker, Clive Brown, Kendra E. Stauffer, Brett W. Petersen, Ryan M. Wallace

**Affiliations:** ^1^Division of High-Consequence Pathogens and Pathology, National Center for Emerging and Zoonotic Infectious Diseases, CDC; ^2^Florida Department of Health; ^3^National IHR Focal Point for Switzerland, Swiss Federal Office of Public Health, Division of Communicable Diseases; ^4^Division of Global Migration and Quarantine, National Center for Emerging and Zoonotic Infectious Diseases, CDC.

On January 16, 2018, CDC was notified by the Florida Department of Health of potential rabies virus exposure in two persons believed to be residents of Switzerland. Rabies virus infections cause a fatal encephalitis, and persons exposed to the virus are advised to receive postexposure prophylaxis (PEP) as soon as possible (*1*). On January 10, 2018, a married couple found a bat in a Naples, Florida, shopping mall parking lot and took it to a local veterinary clinic. The woman, estimated to be aged 50–60 years, stated that they were Swiss tourists. No other identifying information was obtained. On January 15, 2018, the bat tested positive for rabies by the direct fluorescent antibody test at the Florida Department of Health public health laboratory. After repeated efforts to identify the couple were unsuccessful, CDC was able to locate the couple by using the national focal point network maintained by World Health Organization (WHO) International Health Regulations (IHR) (*2*); the two were promptly administered PEP.

After the bat tested positive for rabies, multiple follow-up interviews during the next 3 days with staff members at the local veterinary clinic and mall employees conducted by the Florida Department of Health revealed no additional information regarding the couple. Because of the limited information available about the couple, their potential for a rabies virus exposure, and concern that they might have traveled out of the Naples area, the Florida Department of Health in Collier County next issued a press release on January 18 requesting that the couple contact health department officials.*

CDC worked with the Florida Department of Health from January 16 to January 24 to try to gain more information regarding the couple. On January 24, with no further identifying information obtained and the couple still not located, the CDC Poxvirus and Rabies Branch and the Division of Global Migration and Quarantine contacted the national focal point in Switzerland identified through the IHR network maintained by WHO to inform the Swiss government of the incident. Under article 44 of IHR, WHO encourages international collaborations for the detection and assessment of public health events. IHR national focal points are positions within governments staffed by points of contact who are available at all times to send urgent communications and disseminate information when public health risks of potential international concern are identified (*2*). National focal points regularly share pertinent public health information for contact tracing with each other. Accordingly, CDC contacted the Swiss IHR national focal point because of the need for timely risk assessment and administration of PEP to prevent rabies virus infection.

The Swiss Federal Office of Public Health issued a national press release on January 25 and was contacted by the couple within 5 hours (personal communication, Jesse Blanton, CDC, 2018). The couple confirmed handling the bat with their bare hands but did not report being bitten. The couple also confirmed that no other persons approached the bat during the time it was in their possession. The couple was advised to begin PEP, which was started the same day (January 25).

Since January 2017, CDC’s Poxvirus and Rabies Branch has used the IHR national focal point network to issue health alerts regarding potential rabies virus exposures in 12 persons. The IHR health alerts have resulted in at least four persons receiving potentially lifesaving treatment for rabies (Table).

**TABLE:** Health alerts issued by CDC regarding potential rabies virus exposures using the International Health Regulations National Focal Point Network, January 2017–January 2018

Month	Circumstance	Countries notified	No. of persons exposed	PEP administered
May 2017	Non-U.S. citizens exposed to rabid dog while traveling in India	India, Spain	4	No; public health assessment determined PEP not needed
August 2017	Non-U.S. citizens exposed to bats in Grand Teton National Park, Wyoming, United States	Australia, France, Mexico, United Kingdom	4	Unknown; follow-up is ongoing
January 2018	U.S. citizens exposed to rabid dog imported from Egypt; Egyptian government notified for follow-up investigation	Egypt	2	Yes
January 2018	Non-U.S. citizens exposed to rabid bat in Florida	Switzerland	2	Yes

**Abbreviation:** PEP = postexposure prophylaxis.

The decision to notify the Swiss IHR national focal point was made after considering the severity of rabies virus infections in exposed persons, despite the lack of identifiable information available. This event illustrates the usefulness of the WHO IHR national focal point network for communicating binational, time-sensitive public health information. The Swiss Federal Office of Public Health was rapidly able to employ effective messaging to identify the persons at risk.

The IHR national focal point network maintained by WHO can be activated outside of pandemic situations to strengthen international public health relationships; communicate valuable, time-sensitive information; and strengthen global health capacity through coordinated response efforts. The CDC examples since January 2017 highlight the increasingly multinational nature of rabies virus exposure investigations, the collateral benefit of having the WHO IHR national focal point system in place, and the willingness of IHR signatories to expand its application to urgent situations, even those involving a small number of persons.

## Footnotes

* http://collier.floridahealth.gov/_files/_documents/press-releases/_documents/20180118rabies.pdf.
